# Targeted inhibition of mRNA translation initiation factors as a novel therapeutic strategy for mature B-cell neoplasms

**DOI:** 10.37349/etat.2020.00002

**Published:** 2020-02-29

**Authors:** Joe Taylor, Alison M Yeomans, Graham Packham

**Affiliations:** Cancer Research UK Centre, Cancer Sciences, Faculty of Medicine, University of Southampton, SO16 6YD Southampton, United Kingdom; Istituto Nazionale Tumori “Fondazione Pascale” Via Mariano Semmola, Italy

**Keywords:** mRNA translation, initiation, lymphoma, leukemia, inhibitor, drug

## Abstract

Cancer development is frequently associated with dysregulation of mRNA translation to enhance both increased global protein synthesis and translation of specific mRNAs encoding oncoproteins. Thus, targeted inhibition of mRNA translation is viewed as a promising new approach for cancer therapy. In this article we review current progress in investigating dysregulation of mRNA translation initiation in mature B-cell neoplasms, focusing on chronic lymphocytic leukemia, follicular lymphoma and diffuse large B-cell lymphoma. We discuss mechanisms and regulation of mRNA translation, potential pathways by which genetic alterations and the tumor microenvironment alters mRNA translation in malignant B cells, preclinical evaluation of drugs targeted against specific eukaryotic initiation factors and current progress towards clinical development. Overall, inhibition of mRNA translation initiation factors is an exciting and promising area for development of novel targeted anti-tumor drugs.

## Introduction

Although mRNA translation was viewed for many years as a constitutive, house-keeping function, it is now clear that this process is highly regulated and that its dysregulation can contribute to cancer development. Therefore, targeted inhibition of mRNA translation is a promising new approach to cancer therapy. In this review, we address the biological significance and drug targeting of mRNA translation pathways in mature B-cell neoplasms, a heterogeneous group of cancers that arise from cells of the adaptive immune system. We focus on pathways of B-cell lymphomagenesis/leukemogenesis, regulation of mRNA translation initiation and the effect on new drugs targeted against specific eukaryotic initiation factors (eIFs).

## Mature B-cell neoplasms

Mature B-cell neoplasms arise from B cells which produce protective antibodies in response to foreign antigen. The cell surface B-cell receptor (BCR) plays a key role in determining the function of normal B cells since each clone within the B-cell repertoire has a unique BCR which defines the spectrum of antigens that bind [1]. This BCR diversity is generated by random recombination of immunoglobulin (Ig) gene segments followed by somatic hypermutation of the variable (antigen binding) regions. A diverse BCR repertoire is essential for health since it allows production of antibodies against a broad range of potential antigens. However, colateral effects of these mutagenic processes on non-Ig genes puts B cells at risk of accumulation additional mutations leading to activation of oncogenes/inactivation of tumor suppressors.

**Figure 1.**
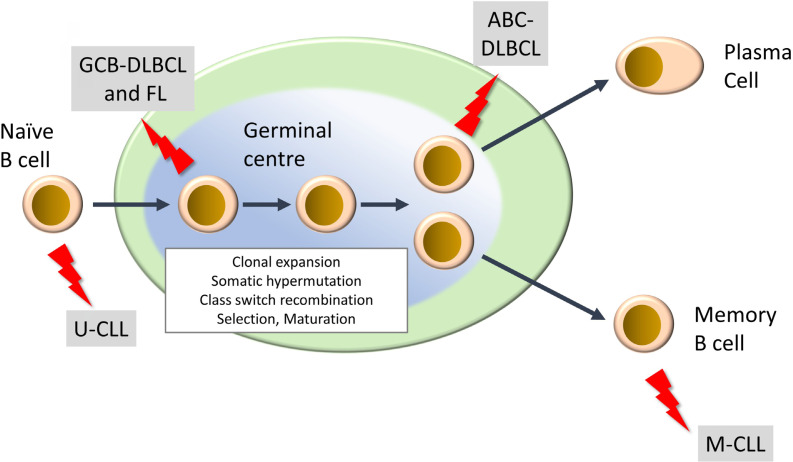
Development of mature B-cell neoplasms. The germinal center (GC) is a key site for development of high affinity antibody responses. It is characterized by clonal expansion of B cells, somatic hypermutation (introduction of new mutations into Ig variable regions) and class switch recombination (where the constant region of the BCR is “switched” from IgM/IgD to other isotypes (IgG, IgA, IgE). There is also intense selection whereby B cells with higher affinity BCRs are positively selected whereas others are deleted by apoptosis. Selected B cells then mature to either antibody-producing plasma cells or memory B cells. “Lightning” symbols indicate transforming events leading to generation of FL and GCB-DLBCL from GC B cells and ABCDLBCL from a slightly more mature B cell termed a plasmablast. CLL comprises two main subsets derived from pre-GC (U-CLL) and post-GC (M-CLL) B cells

Mature lymphoid neoplasms are a strikingly diverse family of malignancies, both in terms of their etiology and clinical behavior, including response to treatment [2–4]. They are typically diagnosed in older individuals and their incidence is rising. There are more than 50 subtypes and this review will focus on the most common. This includes two subtypes of non-Hodgkin’s lymphoma, diffuse large B-cell lymphoma (DLBCL) and follicular lymphoma (FL), of which DLBCL is the most common aggressive B-cell lymphoma and FL is the most common indolent lymphoma. Chronic lymphocytic leukemia (CLL) is the most common B-cell leukemia. DLBCL and CLL can be further subdivided into activated B-cell-like (ABC) and germinal center B-cell-like (GCB) DLBCL, and unmutated CLL (U-CLL) and mutated CLL (M-CLL), respectively, and arise from different stages of B-cell differentiation within secondary lymphoid organs (Figure 1). In some cases, indolent disease can “transform” to a high-grade malignancy. In the UK, the annual incidence rates (per 100,000) for DLBCL, FL and CLL are 8.5, 3.4 and 7.2, respectively (https://www.hmrn.org/statistics/quick).

Due to the risk of off-target effects associated with *Ig* recombination, mature B-cell neoplasms are characterized by a high frequency of chromosomal translocations. For example, the t(14:18) translocation leads to BCL2 overexpression and suppression of apoptosis, and is a hallmark of FL [5]. The t(14:18) translocation is also found in GCB-DLBCL, but overall, the genetic complexity of DLBCL is greater than FL, and additional translocations may be present (e.g., leading to MYC overexpression) [6, 7]. There are also examples of “double hit” or “triple hit” DLBCL which are characterized by co-occurrence of mutations leading to activation of BCL2 with MYC and/or BCL6, and these are associated with a particularly poor outcome [8]. Translocations are much rarer in CLL and here BCL2 overexpression is commonly driven by loss of inhibitory miRNAs associated with recurrent deletion of part of chromosome 13 [9]. In addition to chromosomal changes, B-cell neoplasms are also characterized by somatic mutations affecting a wide range of pathways, including DNA damage (e.g., *TP53*, *ATM*), intracellular signaling pathways (e.g., *MYD88*, *CD79B*, *NOTCH1*, *TNFRSF14*), cell cycle control (*CDKN2A*) and epigenetics (e.g., *EZH2*, *CREBBP*) [2, 6, 7, 10, 11].

There is also an important role of the microenvironment in the development of these malignancies, including potential influences of T cells, stromal cells and cells of the innate immune system [12, 13]. Expression of the BCR is typically retained post-transformation and it is thought to play a major role by engaging pro-growth, proliferation and survival pathways, in response to antigen, environmental lectins or via autonomous BCR:BCR interactions [5, 14, 15].

Treatment for mature B-cell neoplasms typically relies on combinations of cytotoxics with anti-CD20 antibody therapy, for example, fludarabine, chlorambucil and rituximab (FCR) for CLL, and cyclophosphamide, doxorubicin, vincristine, prednisone and rituximab (CHOP-R) for FL/DLBCL [2, 3, 16]. DLBCL may be cured in 60–70% of cases but new treatments are required for a substantial proportion of patients, especially for ABCDLBCL and double/triple-hit DLBCL which have poorer outcomes. FL and CLL are both considered incurable although there may be a proportion of M-CLL cases with very long-term responses to FCR [17]. However, in most cases, initial responses can be promising but these low-grade malignancies typically follow a remitting/ relapsing course. New therapies targeted against cell survival pathways (e.g., venetoclax, a BCL2 inhibitor) or BCR-associated signaling kinases (e.g., ibrutinib and idelalisib, inhibitors of BTK and PI3K, respectively) can result in dramatic clinical responses and have revolutionized treatment in some cases [2, 18–21]. However, development of resistance and toxicity are major issues.

Overall, mature B-cell neoplasms are a heterogeneous group of malignancies which represent a significant and growing burden of morbidity and mortality. Existing treatments can be effective but there is a clear need for new treatment options, particularly for more aggressive disease subsets.

## Mechanisms of mRNA translation

mRNA translation is the process by which the triplet code on mRNA is decoded by the ribosome to produce a polypeptide chain [22]. mRNA translation was considered for many years as a constitutive or “house-keeping” function. However, it is now clear that this process is highly regulated and that the mechanisms controlling mRNA translation are as sophisticated as those controlling transcription. Indeed, studies have indicated that modulation of mRNA translation may play as great a role as modulation of transcription in determining differences in protein expression between cell states [23]. Importantly, translational regulation provides a rapid mechanism to modulate protein expression compared to transcription. mRNA translation is usually considered in three steps, initiation, elongation and termination. mRNA translation initiation requires the action of multiple eIFs and accessory proteins, and is the most highly regulated step.

**Figure 2.**
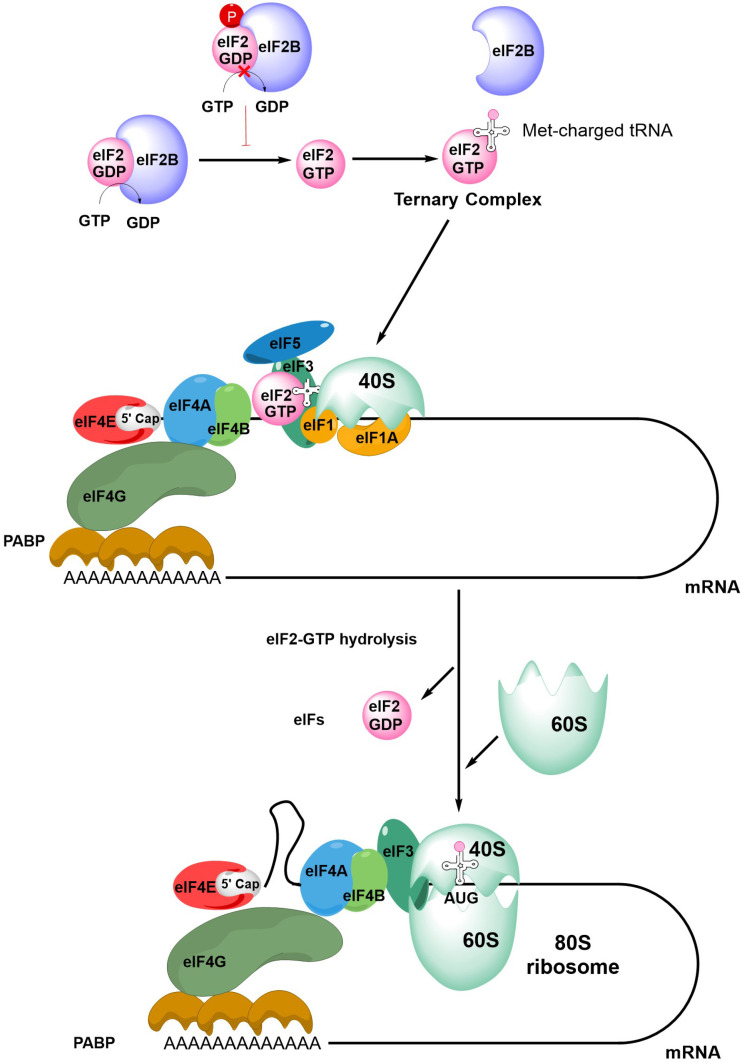
An overview of mRNA translation initiation. The Figure shows some of the key steps in initiation of translation. The process initiates with the joining of the eIF4F complex (eIF4A, eIF4E and eIF4G), the tertiary complex, the 40S ribosome subunit and accessory eIFs. Interactions between eIF4G and PABP lead to closed-loop formation/mRNA circularization. The subsequent steps of scanning, initiation codon recognition and large ribosome subunit joining are accompanied by eIF2-GTP hydrolysis, release of eIF2-GDP and other eIFs. Note figure is illustrational and does not show all intermediate steps. See text for further details

The first step in initiation of mRNA translation is formation of the eIF4F complex which contains eIF4A, eIF4E and eIF4G (Figure 2). eIF4E binds to the m^7^G-cap which is present at the 5′ end of mRNAs whereas eIF4A is a helicase which is important for unwinding mRNA secondary structures. eIF4G is a scaffold protein which binds eIF4A and eIF4E, and also contacts poly(A) binding protein (PABP) bound to the poly(A) 3’-tail of mRNA, thereby forming a circular messenger ribonucleoprotein complex, also known as the “closed-loop” structure. eIF4G/PABP interactions enhance mRNA translation initiation and can also influence recycling of ribosomes, translational termination and mRNA surveillance, for example, by effects on nonsense-mediated decay [24–27].

Formation of the eIF4F:mRNA complex allows the recruitment of the ternary complex (TC) which contains GTP-bound eIF2 (a complex comprising α, β and γ subunits) and the methionine-loaded initiator tRNA. eIF2B is important for mRNA translation initiation since it is a guanine exchange factor required for GTP loading of eIF2α. The TC then binds the 40S ribosome subunit, an interaction that is stabilized by eIF1, eIF1A and eIF3, resulting in formation of the 43S pre-initiation complex (PIC) and binding of eIF5. This complex then “scans” the mRNA until it recognizes an initiation codon (usually AUG) in a suitable sequence context. Start site recognition is followed by arrest of the ribosome and hydrolysis of GTP within the TC. This triggers release of GDP-bound eIF2 and other eIFs and allows binding of the 60S ribosomal subunit to generate the 80S ribosome initiation complex.

Initiation is followed by elongation, whereby rounds of polypeptide bond formation add amino-acids to the nascent polypeptide chain. Finally, recognition of a termination codon leads to termination of mRNA translation and dissociation of the ribosome.

In addition to this cap-dependent mechanism of mRNA translation initiation, some mRNAs can be translated via alternate mechanisms [28]. For example, internal ribosome entry sites (IRES) in the 5′-untranslated region (UTR) of specific mRNAs can interact with canonical eIFs and IRES trans-acting factors to recruit ribosomes independently of the m^7^G-cap. Although originally described in viral mRNAs, IRESs facilitate translation of specific cellular mRNAs, especially in situations of stress, where m^7^G-cap-dependent mRNA translation is often inhibited. Of particular relevance to mature B-cell neoplasms, both the BCL2 and MYC oncoproteins can be translated via IRES-dependent mechanisms under at least some conditions [29, 30].

It is important to note that some eIFs have additional roles in mRNA-related processes. In particular, the m^7^G-cap binding function of eIF4E is exploited in the export of some mRNAs from the nucleus [31]. eIF4E’s role in RNA export is dependent on XPO1 (CRM1), which is itself required for export of multiple cargoes, including many proteins [32].

## Selectivity and regulation of mRNA translation initiation

Variation in mRNA translation makes a major contribution to determining the differences in the proteome between cell types and cell states. Regulatory mechanisms influence overall translational capacity or can act on specific mRNAs to control expression of individual proteins. The expression of many proteins which are particularly relevant to biology of neoplastic B cells are subject to tight translational control, include regulators of cell growth (MYC), cell cycle progression (cyclin D1), and suppression of apoptosis (MCL1, BCL2). These are discussed in more detail below.

Some of the regulatory mechanisms that influence mRNA translation depend on intrinsic features present within the 5′-UTRs of target mRNAs that may be “sensed” by components of the eIF4F complex. For example, the helicase activity of eIF4A appears to be particularly important to unwind and facilitate translation of mRNAs with highly structured 5′-UTRs [33–36]. This includes mRNAs containing G-quadruplexes, RNA structures comprising stacked G-quartets stabilized by non-Watson-Crick interactions [33], which are predicted to occur in the 5′-UTRs of a substantial proportion of mRNAs, including BCL2 [37, 38]. These highly stable RNA structures are generally inhibitory for mRNA translation, either by preventing 43S PIC binding to mRNA or slowing scanning [39, 40].

Like eIF4A, eIF4E-dependency has also been linked to the presence of secondary structure but this may reflect the requirement of eIF4E for recruitment of eIF4A to the eIF4F complex. However, some specific mRNA 5′-UTR sequences have been linked to increased eIF4E-dependency. This includes so-called 5′-terminal oligopyrimidine (TOP) mRNAs which contain a cytidine residue at the m^7^G-cap followed by an uninterrupted sequence of pyrimidines [41]. Translation of these mRNAs is particularly sensitive to inhibition of mTORC1, a major upstream regulator of eIF4E activity [42, 43] (see below). However, not all mTORC1 sensitive mRNAs contain TOP sequences, and other specific sequence elements have also been linked to eIF4E dependency [43–45]. In mouse models, eIF4E haploinsufficiency is compatible with normal development but not for transformation, providing a potential explanation for the frequent activation of eIF4E in cancer cells [46]. Interestingly, mRNAs whose translation was particularly sensitive to eIF4E haploinsufficiency were frequently characterized by the presence of a 15 nucleotide 5′-UTR motif termed the cytosine enriched regulator of translation (CERT).

In addition to a requirement for the m^7^G-cap, dependency on eIF4E for mRNA nuclear export is also determined by specific mRNA sequences, specifically a ∼50-base 3’-UTR sequence element, known as 4E sensitivity element (4E-SE) [47]. mRNAs containing 4E-SE include *CCND1*, *MYC* and *ODC1* and are particularly dependent on eIF4E for nuclear export.

Finally, it is important to note that several eIFs exist as multiple isoforms which may be functionally distinct. eIF4E2 was not thought to play a major role in mRNA translation initiation due its weaker m^7^G-cap and eIF4G binding activity, relative to eIF41. However, more recent data indicates that eIF4E2 may play a key role in sustaining mRNA translation in hypoxia [48]. Moreover, although there is considerable overlap in the mRNAs which are particularly sensitive to levels of eIF4E1 and eIF4E3, these factors are also associated with preferential translation of specific mRNAs via distinct 5′-UTR sequence motifs [49].

mRNA dependent translational control is also mediated by miRNAs which typically bind to sequences within the 3’-UTRs of target mRNAs [50]. Although miRNAs can induce degradation of their target mRNAs, their principle mode of action is via translational repression and some studies have indicated that the mechanism of miRNA-mediated translational repression is dependent on the eIF4A isoform eIF4A2 [51].

Various miRNAs have been linked to malignant features of B-cell neoplasms. As described above, deletion of part of chromosome 13 in CLL cells results in loss of miR-15a and miR-16-1, and subsequent suppression of apoptosis by increased BCL2 expression [9]. Other examples reveal the importance of complex networks linking B-cell neoplasm-associated proteins/pathways with multiple miRNAs [52]. For example, BCR signaling in CLL cells drives a malignancy promoting pathway mediated by increased expression of the miR-132-3p/miR-212 cluster, which in turn repress expression of TGFB1 and the negative cell cycle regulators EP400 and ZBTB5 [53]. In turn, BCR signaling is modulated by various miRNAs, including miR-155, miR150 and miR-17-92 [54–56]. There is also strong evidence that MYC is a central node in miRNA circuitry, since its expression is modulated by miRNAs (e.g., miR-34a and let-7) and it is itself a regulator of miRNA expression [57, 58].

**Figure 3.**
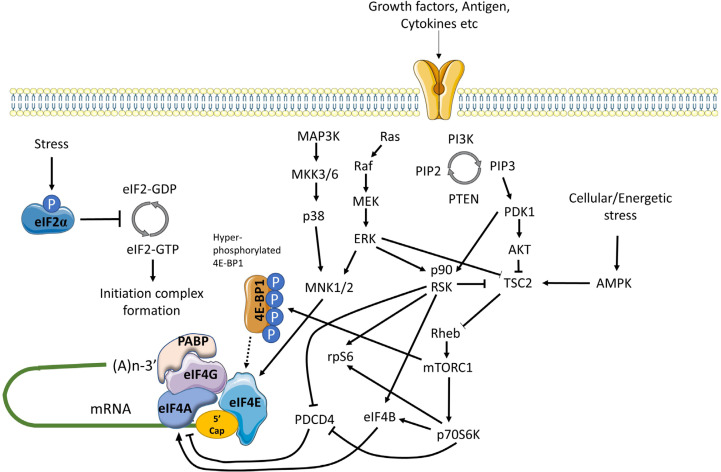
Positive and negative regulation of mRNA translation initiation by signaling pathways. mRNA translation is promoted by PI3K/AKT/mTORC1 and MAPK signaling. mTORC1 is activated via repression of TSC2-mediated conversion of Rheb-GTP to inactive Rheb-GDP. mTORC1 promotes the hyper-phosphorylation of 4E-BP1 which prevents inhibitory association with eIF4E, facilitating eIF4F complex formation. mTORC1 additionally activates p70S6K which indirectly activates eIF4A via repression of PDCD4 and activation of eIF4B. MAPK signaling leads to activation of MNK1/2 (and downstream phosphorylation of eIF4E) via MEK/ERK and p38 pathways, and ERK which with downstream-activated p90RSK, also relieves TSC2 mediated repression of mTORC1. In response to energetic stress AMPK is activated which promotes TSC2 mediated conversion of Rheb-GTP to Rheb-GDP to inhibit mTORC1. Other stress stimuli promote eIF2α phosphorylation that arrests the eIF2 complex in its inactive GDPbound form repressing mRNA translation

mRNA translation is also controlled by multiple signaling inputs commonly activated downstream of cell surface receptors (Figure 3) [59]. These pathways can act globally to modulate overall mRNA translational capacity and/or influence translation of specific mRNAs to exert protein-specific control.

One of the key kinase complexes controlling mRNA translation is mTORC1 which comprises the mTOR kinase and various accessory proteins (raptor, GβL and deptor) [60]. The principal mTORC1 substrates for translational regulation include 4E-BP1 and p70S6K. Hypophosphorylated 4E-BPs interfere with eIF4F assembly by binding to a site on eIF4E that overlaps the eIF4E:eIF4G interaction interface. Thus, mTORC1-induced 4E-BP1 phosphorylation relieves inhibition of eIF4E and results in increased mRNA translation, including of TOP mRNAs [42, 43]. The function of p70S6K is less well understood, but its substrates include ribosomal protein S6 and eIF4B, an auxiliary factor that enhances eIF4A helicase activity. It can also phosphorylate and induce proteosomal degradation of PDCD4, an endogenous inhibitor of eIF4A [61].

mTORC1 activity is regulated by the TSC complex which inhibits mTORC1 activity by converting Rheb-GTP (an activator of mTORC1) into its inactive GDP-bound form [62]. Activation of AKT following receptor stimulation results in inhibitory phosphorylation of TSC2 (a component of the TSC complex), thereby relieving TSC-mediated repression of mTORC1 activity.

mRNA translation is also regulated by MAP kinase pathways, especially via ERK1/2 and p38 which can phosphorylate and activate MNK1/2 kinases [63]. Once activated, MNK1 phosphorylates eIF4E on Ser^209^ which appears to enhance its activity for both mRNA translation initiation and nuclear export. MNKs may also regulate mRNA translation initiation by phosphorylation of eIF4G. Interestingly, in DLBCL, MNK1 and MNK2 expression differs between the GCB and ABC subsets and this results in differential translation of a subset of mRNAs via effects on eIF4E1 and eIF4E3 isoforms [49].

Finally, there is cross-talk between the AKT/mTORC1 and MAPK pathways that influence mRNA translation. Perhaps most importantly, ERK1/2, and downstream p90RSK can promote inhibitory phosphorylation of TSC2, thereby contributing to activation of mTORC1 following receptor stimulation [59]. p90RSK can also phosphorylate rpS6 on Ser^235/236^ [59] and regulate eIF4A activity through phosphorylation of PDCD4 and eIF4B [64–66].

Cell stress can lead to a profound repression of mRNA translation, principally via an integrated stress response mediated by induced phosphorylation of eIF2α on Ser^51^ [67]. eIF2α Ser^51^ phosphorylation prevents guanine exchange by eIF2B, trapping eIF2 in its inactive GDP-bound form. eIF2α phosphorylation is catalyzed by one of four kinases PERK (activated following accumulation of unfolded proteins in the endoplasmic reticulum), PKR (activated in response to dsRNA during virus infection), HRI (activated by heme depletion, oxidative stress and heat-shock), and GCN2 (activated by amino-acid starvation). Whilst eIF2α phosphorylation is associated with profound repression of mRNA translation, translation of some mRNAs escapes this control allowing maintenance of protein expression (e.g., ATF4).

mRNA translation is extremely energy consuming and there are multiple mechanisms to ensure close coupling between mRNA translation capacity and ATP levels, and also with availability of amino-acids. Thus, amino-acid depletion negatively regulates mTORC1 activity via multiple mechanisms [68]. Moreover, a reduction in ATP availability leads to activation of AMPK which phosphorylates activatory sites on TSC2 leading to mTORC1 inactivation [60].

Finally, translational activity can be modulated by transcriptional control of its regulators. A key example is MYC which increases expression of mRNAs encoding eIF4A, eIF4E and eIF4G, as well as many other components involved in mRNA translation (e.g., ribosome components) [69, 70]. *MYC* mRNA translation is itself subject to tight translational control, suggesting the operation of a positive feedback loop. Another important factor that regulates mRNA translation via effects on transcription is the tumor suppressor p53 which represses expression of eIF4E [71].

## Evidence linking alterations in mRNA translation initiation to mature B-cell neoplasms

Several studies have demonstrated that the expression of some eIFs/translational regulators differ between subtypes of mature B-cell neoplasms or between malignant B cells and their normal B-cell counterparts. Wang et al.[72], used immunohistochemistry of lymph node samples to demonstrate that expression of eIF4E and eIF2α was higher in more aggressive lymphoma subtypes compared to indolent subtypes. A subsequent study demonstrated that eIF4E expression in DLBCL was associated with poor outcome in both ABC and GCB subtypes [73]. Kodali et al.[74], demonstrated that 4E-BP1 expression was more commonly detectable in mature neoplasms (including FL, DLBCL and small lymphocytic lymphoma (SLL), the tissue-based counterpart of CLL) compared to non-malignant samples, whereas levels of 4E-BP1 phosphorylation were more variable between samples. Immunohistochemistry analysis also demonstrated that expression of the eIF4GII isoform was increased in DLBCL cells compared to normal B cells [75]. This may be linked to reduced expression of miR-520c-3p, a negative regulator of eIF4GII expression in DLBCL cells.

More recent studies using quantitative PCR demonstrated that 12/16 eIFs surveyed were overexpressed in DLBCL samples compared to normal controls [76]. Overexpression of eIF1A1, eIF3D and eIF2B5 was observed in both the GCB and non-GCB-DLBCL subtypes, but was somewhat greater in non-GCB-DLBCL, and was confirmed by immunohistochemistry. Interestingly, higher levels of eIF2B5, which is part of the eIF2B complex required for GTP loading of eIF2α, was associated with poor survival at both the RNA and protein level.

eIF4B (an activator of eIF4A) has been shown to be overexpressed in DLBCL cells compared to normal controls, and higher eIF4B expression was associated with poorer outcome [77]. This increased eIF4B expression was associated with enhanced translation of mRNAs encoding proteins involved in suppression of apoptosis and DNA repair, including BCL2, DAXX and ERCC5. One novel potential lymphoma-associated driver of eIF4B is fatty acid synthase (FASN) which stabilizes eIF4B and increase expression of oncoproteins such as MYC, BCL6 and MCL1 (a BCL2-related anti-apoptotic protein) via formation of a complex between eIF4B and the deubiquitinase USP11 in DLBCL cells [78].

Mouse models have also provided strong evidence that dysregulation of mRNA translation initiation contributes to lymphomagenesis. In particular, eIF4E co-operates with MYC to promote lymphomagenesis [79, 80]. Moreover, eIF4E overexpressing-lymphomas are relatively resistant to chemotherapy demonstrating that dysregulated mRNA translation can influence both tumor development and treatment responses [81]. More recent mechanistic analysis demonstrated that the ability of eIF4E to cooperate with MYC was dependent on the ability of eIF4E to bind the m^7^G-cap, but possibly not promote mRNA transport, at least in these models [82]. Interestingly, eIF4E activity was dependent on MNK1/2-mediated phosphorylation since a non-phosphorylatable mutant of eIF4E was inactive in these assays. Consistent with this, activated MNK1 also cooperated with MYC to promote lymphomagenesis. The anti-apoptotic MCL1 protein was identified as a key effector of this MNK/eIF4E pathway. Further linkage between mRNA translation initiation and lymphomagenesis is provided by observation that the deubiquitinase UCH-L1, which promotes assembly of eIF4F, is required for MYC-induced lymphomagenesis [83].

eIF6 plays a role in both ribosome biogenesis and mRNA translation initiation and seems to contribute to malignant transformation of B cells, since a reduction in eIF6 expression by deletion of single *eIF6* allele is sufficient to reduce MYC-induced lymphomagenesis [84]. Interestingly, eIF6^−/+^ mice are healthy, indicating that malignant cells are particularly sensitive to the abundance of eIF6. eIF6 promotes glycolysis and fatty acid synthesis via increased translation of a subset of target mRNAs characterized by 5′-UTRs with a high G:C content or upstream open reading frames (both of which are generally inhibitory to translation) and reprogramming of metabolism by eIF6 may be required to support MYC-induced lymphomagenesis [84]. This critical dependency on eIF6 levels for transformation is reminiscent of eIF4E where haploinsufficiency is also sufficient to substantially reduce susceptibility to oncogene-induce transformation but is seemingly well tolerated by normal cells [46]. These are important observations since it suggests that may be possible to therapeutically inhibit eIF6 or eIF4E function in malignant cells with relatively little effect on normal cells.

Deletion of the eIF4A inhibitor PDCD4 has also been shown to increase lymphomagenesis in the mouse [85]. However, PDCD4 may have additional functions beyond translational control [86] and it is not clear to what extent increased eIF4A activity might contribute to increased lymphomagenesis in PDCD4-deficient animals.

Next generation sequencing has revealed the presence of recurrent mutations of genes encoding several factors related to mRNA translation, including XPO1, DDX3X, RPS15 and EIF2AK3 (PERK) in mature B-cell neoplasms [6, 7, 11]. Recurrent *XPO1* mutations have been described in CLL and DLBCL, most commonly leading to amino-acid substitutions at E571. E571-mutant XPO1 co-operates with BCL2 or MYC to promote lymphomagenesis *in vivo* and is associated with altered nuclear/cytoplasmic distribution of various protein cargoes [87]. Mutant XPO1 also sensitized cells to the effects of the XPO1 inhibitor selinexor both *in vitro* and *in vivo*. However, the effects of XPO1 mutations on eIF4E-mediated mRNA transport have not been analyzed.

DDX3X is an RNA helicase that appears to play a specialized role in mRNA translation initiation, including non-AUG-initiated translation [88, 89]. Recurrent truncating mutations, leading to reduced DDX3X protein expression, have been described in CLL and DLBCL [90]. *DDX3X* mutations have also been described in medulloblastoma and more detailed functional analyses have shown that these mutations are associated with reduced global mRNA translation [91]. However, it is important to note that the spectrum of mutations of *DDX3X* differs between B-cell neoplasms and medulloblastoma (truncating mutations versus nonsynonymous single nucleotide changes) and the functional consequences of DLBCL/CLL-associated *DDX3X* mutations have not been reported.

Overall, several factors related to mRNA translation have been shown to be recurrently mutated in mature B-cell neoplasms and these may contribute to pathogenesis and influence drug responses. However, it is important to note that in the most common forms of mature B-cell neoplasms these mutations are found in a relatively small proportion (typically < 10%) of patients and most cases therefore do not appear to have mutations that directly dysregulate mRNA translation. However, there are some notable exceptions. For example, the frequency of *XPO1* mutations may reach 50% in primary mediastinal B-cell lymphoma (a rare form of B-cell lymphoma) [92].

In the absence of mutations directly affecting the translational machinery, it seems likely that inappropriate mRNA translation in mature B-cell neoplasms is driven by upstream signaling pathways, activated by mutation or microenvironmental stimulation. Significant mutational drives likely include activation of mTORC1 signaling, e.g., due to loss of PTEN (a negative regulator of PI3K activity) in DLBCL or acquisition of mutations affecting *RRAGC*, which encodes an upstream activator of mTORC1, in FL [6, 7, 93]. Indeed, mTORC1 activity is required to drive the increased expression of eIF4B that is a feature of DLBCL [77]. In CLL, truncating mutations leading to activation of NOTCH1 signaling have been shown to be associated with increased BCR-induced mRNA translation [94], potentially mediated by a stimulatory effective of NOTCH1 signaling on the MNK/phospho-eIF4E pathway [95].

In terms of microenvironmental stimulation, it is likely that activation of the BCR plays a key role in driving mRNA translation in malignant B cells. Indeed, cross-linking of the BCR of CLL cells *in vitro* leads to increased global mRNA translation as well as translation of the *MYC* mRNA [96]. Moreover, gene expression analysis revealed that CLL cells derived from the lymph nodes of patients (the likely site of BCR stimulation of CLL cells by antigen *in vivo*) have increased expression of gene signatures related to mRNA translation compared to CLL cells in the blood. BCR-induced mRNA translation was associated with increased expression of eIF4A and eIF4G in CLL but not normal B cells, thereby revealing potential differences in mechanism of mRNA translational control between normal and malignant B cells.

In addition to eIFs, BCR signaling down-regulates expression of the eIF4A inhibitor PDCD4 [96, 97]. PDCD4 may be a common link between signaling and mRNA translational control since PDCD4 expression is downregulated following exposure of CLL cells to other microenvironmental signals, including the combination of bone marrow-derived stromal cells, CD40 ligand and CpG oligodeoxynucleotides (which stimulate toll-like receptor signaling), or the chemokine CXCL12 [98, 99].

CD40 ligand/IL4 signaling (to mimic T-cell help for B cells) has also been shown to increase eIF4E expression and 4EBP1 phosphorylation, and global mRNA translation in primary CLL cells [100]. Interestingly, this study also used ribosome profiling to show that several genes encoding proteins involved in the DNA damage response, including ATM, were particularly sensitive to translational control and that CD40 ligand/ IL4 treatment improved DNA damage responses in CLL cells exposed to X-radiation. Importantly, lower levels of ATM expression were associated with poorer outcome.

Finally, it is noteworthy that expression of many proteins with key roles in pathogenesis of mature B-cell neoplasms have been shown to be regulated at least in part at the level of mRNA translation (Table 1). Thus, dysregulated mRNA translation has potential to impinge widely of critical lymphoma/leukemiapromoting pathways.

**Table 1.** Examples of translationally regulated genes with relevance to mature B-cell neoplasms

**Gene(s)**	**Protein function**	**Association with B-cell neoplasms**	**Evidence for translational control via eIFs**	**Refs**
*MYC*	Cell growth	Translocations are a hallmark of Burkitt’s lymphoma Recurrent mutations in double/triple hit DLBCL Increased expression following BCR stimulation is associated with increased mRNA translation	Inhibition of eIF4A reduces MYC expression in B-cell lymphoma cell lines Inhibition of eIF4E reduces *MYC* mRNA translation and nuclear export in DLBCL cell lines	[96, 101–104]
*MCL1*	Cell survival (anti-apoptotic)	Expression induced following BCR stimulation	Inhibition of eIF4A reduces MCL1 expression in B-cell lymphoma cell lines	[103]
*BCL2*	Cell survival (anti-apoptotic)	Translocations are a hallmark of FL Recurrent translocations in GCB-DLBCL and double/triple hit DLBCL	Inhibition of eIF4E reduces *BCL2* mRNA translation and nuclear export in DLBCL cell lines	[101]
*CCND1*	Cell cycle progression	Translocations are a hallmark of mantle cell lymphoma	Inhibition of eIF4A reduces cyclin D1 expression in B-cell lymphoma cell lines	[103]
*BCL6*	Transcriptional repression	Recurrent translocations in double/triple hit DLBCL	Inhibition of eIF4E reduces *BCL6* mRNA translation and nuclear export in DLBCL cell lines	[101]
*CARD11*, *BCL10*, *MALT1*	CBM complex components which control NF-κB activation	Recurrent mutations in subset of ABC-DLBCL Mediates NF-κB activation downstream of BCR	Inhibition of eIF4A reduces CBM complex expression in DLBCL cell lines	[102]

## *In vitro* and *in vivo* effects of eIF inhibition

Consistent with the idea that dysregulated mRNA translation plays a key role in mature B-cell neoplasms, numerous preclinical studies have demonstrated anti-lymphoma/leukemia effects of inhibitors targeted against eIFs.

Analysis of eIF4A inhibitors has focused on naturally occurring flavaglines, particularly silvestrol which was originally isolated from the plant genus *Aglaia*. Silvestrol acts as a chemical inducer of dimerization by forcing interaction between free eIF4A (i.e., not complexed within eIF4F) and mRNA [105, 106] and preferentially reduces translation of mRNAs with highly structured 5′-UTRs [33, 34]. The closely structurallyrelated compound rocaglate A similarly “clamps” eIF4A to mRNAs, particularly those with polypurine tracts within their 5′-UTRs [107]. CRISPR/Cas9 editing has provided strong evidence that anti-cancer activities of flavaglines are mediated via eIF4A1 [108].

Using adoptive transfer of established *Pten*^+/−^*Eμ-Myc* and *Eμ-Myc/eIF4E* lymphoma cells, Bordeleau et al.[105], demonstrated that silvestrol was inactive when tested alone, but did enhance anti-lymphoma activity of doxorubicin. Interestingly, silvestrol did not show anti-lymphoma activity (alone or with doxorubicin) in an *Eμ-Myc/Bcl2* model, suggesting that eIF4A inhibition specifically counters the *in vivo* growth of malignant B cells with a strong drive for translational dysregulation (i.e., either by activation of mTORC1 signaling downstream of PTEN loss or by enforced expression of eIF4E) but not suppression of apoptosis per se by BCL2. However, silvestrol alone has been shown to reduce lymphomagenesis in *Eμ-Myc* mice, where the latency of disease is relatively slow compared to adoptive transfer models [109].

Lucas et al.[110], investigated *in vitro* effects of silvestrol using primary CLL cells and demonstrated that silvestrol reduced expression of *MCL1* protein and induced apoptosis of these cells (IC_50_ < 10 nM). Effects on MCL1 were independent of changes in *MCL1* mRNA levels suggesting that silvestrol’s effects were mediated via translational inhibition. Silvestrol also improved survival in an *Eμ-TCL1* mouse model, which recapitulates features of aggressive CLL.

Schatz et al.[103], demonstrated that silvestrol overcame protective effects associated with overexpression of the PIM2 kinase in an *Eμ-Myc/Tsc2*^−/−^ *in vivo* lymphomagenesis model. Moreover, silvestrol induced apoptosis of various established B-lymphoma cell lines *in vitro* and down-modulated expression of cyclin D1, MYC and MCL1. Steinhardt et al.[102], demonstrated that silvestrol reduced expression of components of the CBM complex (an upstream regulator of NF-κB) in B-lymphoma cell lines following BCR stimulation whereas Wilmore et al.[104], showed that silvestrol reduced induction of MYC protein in primary CLL cells following BCR stimulation. Silvestrol has also been shown to induce apoptosis in various “MYC-driven” B-lymphoma cell lines [111] and this response was associated with reduced translation of *MYC* mRNA. This study also demonstrated that silvestrol synergized with the BCL2 inhibitor ABT-199 to kill lymphoma cell lines *in vitro.*

Cencic et al.[112], studied the alternate eIF4A inhibitor hippuristonal which was originally isolated from the coral *Isis hippuris*. Hippuristonal has a distinct mechanism of action compared to silvestrol and prevents both free and eIF4F-bound eIF4A from interacting with mRNA. Similar to silvestrol, hippuristonal had little activity alone in adoptive transfer models of MYC-induced lymphomagenesis, but cooperated with chemotherapeutics (doxorubicin or cyclophosphamide) to improve survival [113]. Interestingly, antilymphoma activity of hippuristonal was blocked by overexpression of the anti-apoptotic proteins BCL2 or MCL1 and hippuristonal synergized with the BCL2 inhibitor ABT-737 to induce of apoptosis of MYC lymphoma cells *in vitro*.

Other studies have focused on more recently identified eIF4A inhibitors, or on the development of synthetic analogues to overcome liabilities associated with natural product eIF4A inhibitors [111, 114, 115]. For example, silvestrol is a substrate for drug efflux pumps and this dramatically reduces oral bioavailability [116].

Chen et al.[117], recently reported synthetic derivatives of pateamine A which was originally isolated from the sea sponge *Mycale* sp. and like silvestrol, appears to promote association between eIF4A and mRNA. The derivative DMDAPatA induced apoptosis of CLL cells and synergized with ABT-199 to enhance cell killing. Some synthetic pateamine A analogues were shown to have reduced human plasma protein binding, a potential liability associated with the parental compound.

Peters et al.[118], used target-based screening approach to identify the natural product elatol (isolated from the red alga *Laurencia microcladia*) as an inhibitor of eIF4A1. *In vitro* analysis demonstrated that elatol reduced global mRNA translation and reduced expression of MYC, cyclin D3, MCL1, BCL2 and PIM2 in DLBCLderived cell lines. Like silvestrol, elatol also reduced induction of MYC in primary CLL cells following BCR stimulation and reduced the growth of B-lymphoma cells *in vivo*.

The pharmaceutical company eFFECTOR has reported a novel eIF4Ai (eFT226) which has shown promising *in vitro* and *in vivo* preclinical activity in models of lymphoma [119]. It has broad *in vitro* apoptosisinducing activity against cell lines derived from GCB-DLBCL, ABC-DLBCL and mantle cell lymphoma (a less common mature B-lymphoma) and reduces expression of MYC and MCL1. eFT226 also has strong *in vivo* activity against various B-lymphoma lines grown as xenografts in immunocompromised mice, even using a once-weekly dosing schedule.

The synthetic nucleoside ribavirin is used routinely as an anti-viral but has also received substantial attention as an eIF4E inhibitor. Ribavirin mimics the structure of the m^7^G-cap and competes with binding of the m^7^G-cap to eIF4E thereby inhibiting both mRNA translation initiation and nuclear mRNA export [31, 120].

Culjkovic-Kraljacic et al.[101], investigated the effect of ribavirin in models of double- or triple-hit DLBCL. They showed that ribavirin reduced the nuclear export and translation of *BCL6*, *MYC* and *BCL2* mRNAs. Importantly, these effects were could be observed at concentrations of ribavirin (10 μM) that were similar to the steady state concentration of drug found in the plasma of patients undergoing anti-viral ribavirin treatment. Ribavirin reduced growth *in vivo* of a patient-derived xenograft derived from a triple hit DLBCL. Interestingly, ribavirin cooperated with an HSP90 inhibitor to suppress tumor growth (patient-derived lymphoma xenograft or xenografts of established double/triple-hit B-cell lines) potentially since HSP90 binds to eIF4E and may be required to maintain or enhance its activity. In CLL cells, ribavirin sensitizes to pro-apoptotic effects of fludarabine [121].

eFFECTOR has described a series of eIF4E inhibitors which compete for binding to the m^7^G-cap [122]. Ribosome profiling reveals that these compounds preferentially inhibit the translation of mRNAs containing TOP or CERT elements. Screening of a panel of human cell lines showed that cell lines derived from various hematological malignancies were particularly sensitive to growth inhibitory effects of these compounds, and that they also had substantial *in vivo* activity against the TMD8 (ABC-DLBCL-derived) cell line.

Small molecules have also been used to explore the effect of disruption of the eIF4:eIF4G interaction on malignant B cells. Cencic et al.[123], used molecular modelling and ultra-high throughput screening to identify 4E1RCat as an eIF4E:4G1 interaction inhibitor and showed that this compound effectively inhibited m^7^G-cap-dependent mRNA translation initiation and expression of MYC and MCL1 in Jurkat T-leukemia cells. 4EIRCat also cooperated with doxorubicin to extend survival in mice following adoptive transfer of *Pten*^+/−^ *Eμ-Myc* lymphoma cells. Willimott et al.[124], demonstrated that a distinct eIF4E:4G1 interaction inhibitor, 4EGI-1, reduced translation of *MCL1*, *BCL2A1* and *BCL2L1* mRNAs, which encode anti-apoptotic BCL2-related proteins [125]. Moreover, 4EGI-1 and ABT-737 cooperated to promote CLL cell apoptosis.

Given the tight regulation of mRNA translation initiation by signaling, various kinase inhibitors have been used to examine the effects of indirect inhibition of eIFs by targeting of upstream activatory signaling. Most of this work has focused on inhibitors of mTORC1, especially rapamycin or related rapalogues (e.g., everolimus, temsirolimus). These compounds promote formation of a complex between mTORC1 and the immunophilin FKBP12 thereby reducing mTORC1 activity, whilst having relatively little effects on function of mTORC2 (an alternate mTOR kinase-containing complex), at least in short term experiments. Numerous studies have demonstrated ability of rapalogues to reduce proliferation or induce apoptosis of malignant B cells [126–129].

Despite this encouraging preclinical data, rapalogues have only been approved for treatment of mantle cell lymphoma and clinical responses in other B-cell neoplasms are generally modest [130–133]. The reasons for this modest clinical efficacy is not fully understand but may be due to incomplete inhibition of 4E-BP1 phosphorylation and/or stimulation of feedback loops leading to AKT activation which may be mediated via mTORC2 [134]. Thus, more recent efforts have focused on development of direct mTOR kinase inhibitors, which inhibit activity of both the mTORC1 and mTORC2 complexes, and dual mTOR/PI3K inhibitors [135, 136]. Such compounds have also demonstrated strong activity against malignant B cells in preclinical models, and in some cases have been shown to be superior to rapalogues [73, 137–139].

Given their important role in translational control, MNK inhibitors also offer considerable promise as potential therapies for B-cell neoplasms. eFFECTOR recently described the novel dual MNK1/2 inhibitor eFT508 and demonstrated that this compound inhibits the growth of a subset of DLBCL cell lines *in vitro* and has *in vivo* anti-lymphoma activity versus ABC-DLBCL xenografts [140, 141]. Consistent with MNK1/2 inhibition, eFT508 reduced eIF4E phosphorylation *in vitro*. Wu et al.[142], recently described QL-X-138, a dual inhibitor of MNKs and BTK, a pivotal kinase activated downstream of the BCR. QL-X-138 induced greater levels of apoptosis in primary CLL cells compared to ibrutinib or the MNK inhibitor cercosporamide alone.

Although not directly targeted towards eIFs, it is also worth considering inhibitors of XPO1 which is required for eIF4E to mediate its effects on nuclear export of mRNAs. Selective inhibitors of nuclear export (SINEs), including KPT-185, KPT-251 and selinexor, induce apoptosis of CLL cells *in vitro*, including samples with p53 dysfunction, and overcome the survival-promoting effects of microenvironmental stimuli [143]. Despite the importance of XPO1 in many cellular functions, normal B cells appear to be less sensitive to SINEinduced apoptosis compared to CLL cells. These compounds also have *in vivo* anti-leukemia activity in the *Eμ-TCL1* model. Although SINEs have been shown to reduce protein nuclear export, effects on mRNA export and/or translation per se have not been investigated in these models. Selinexor also synergizes with ibrutinib to induce apoptosis of CLL cells *in vitro* and to improve anti-tumor activity *in vivo* in the *Eμ-TCL1* model [144]. Moreover, selinexor retains activity in models of ibrutinib resistance.

## Clinical observations

Clinical development of mRNA translation inhibitors has been led by the elongation inhibitor homoharringtonine (omacetaxine mepesuccinate) which is approved for third-line treatment of chronic myeloid leukemia [145]. Homoharringtonine activity is thought to be dependent on inhibition of translation of the BCR-ABL fusion protein which is critical for development of this leukemia.

The most advanced area of clinical testing related specifically to inhibition of mRNA translation initiation is for signaling inhibitors, especially targeted against mTORC1. As described above, promising preclinical data obtained with rapalogues has not resulted in substantial clinical progress, except for mantle cell lymphoma where temsirolimus is approved for treatment of relapsed/refractory disease. Moreover, the clinical benefit of next generation inhibitors, including dual mTORC1/2 and mTORC1/PI3K inhibitors, remains unclear [134, 146, 147]. Future clinical studies should focus on combinations to boost responses and identification of biomarkers which could reveal subset of responsive patients. For example, recent studies have shown that lymphoma-associated *RRAGC* mutations may confer enhanced sensitivity to mTORC1 inhibition [148].

The dual MNK1/2 inhibitor eFT508 is currently undergoing clinical evaluation as part of a phase 1/2 trial in patients with previously treated lymphoma (NCT02937675) [149].

The SINE selinexor has been approved for treatment of refractory multiple myeloma (derived from plasma cells) and has been evaluated for clinical activity in mature B-cell neoplasms. A phase 1 trial (NCT01607892) in patients with advanced hematological malignancies reported an overall objective response rate of 31%, although this was accompanied by significant toxicity [150]. On the basis of several complete responses, selinexor is currently being evaluated in a phase 2 trial in relapsed/refractory DLBCL (NCT02227251). Selinexor in combination with ibrutinib is also being evaluated in a phase 1 trial in patients with relapsed/ refractory CLL or aggressive NHL (NCT02303392). Other XPO1 inhibitors are in preclinical development and may offer a wider therapeutic window compared to selinexor [151].

Several case reports have described possible anti-lymphoma responses in individuals treated with ribavirin (alone or in combination with other anti-virals) for viral infection/reactivation [152, 153]. This includes a patient initially diagnosed with CLL that had undergone transformation to Hodgkin’s lymphoma who showed near complete response of lymph node lesions following treatment with ribavirin in the absence of any anti-lymphoma treatment [152]. It is notable that ribavirin has shown clinical activity in acute myeloid leukemia [154] and a trial to determine potential clinical activity of ribavirin in indolent FL and mantle cell lymphoma is underway (NCT03585725).

The eFFECTOR eIF4Ai (eFT226) is currently being evaluated for clinical activity in advanced solid tumor malignancies (NCT04092673) but will also be a very interesting candidate for clinical testing for hematological malignancies.

## Conclusions

There has been a major shift in our perspective on mRNA translation, from a constitutive, house-keeping function to a highly regulated and selective process that is frequently dysregulated in cancer. In parallel, there has been a growing focus on targeted inhibition of mRNA translation as a novel strategy for cancer therapy. The field has progressed rapidly, and we now have much deeper insight into mechanisms of regulation and dysregulation of mRNA translation, and promising new chemical agents to target these pathways.

**Figure 4.**
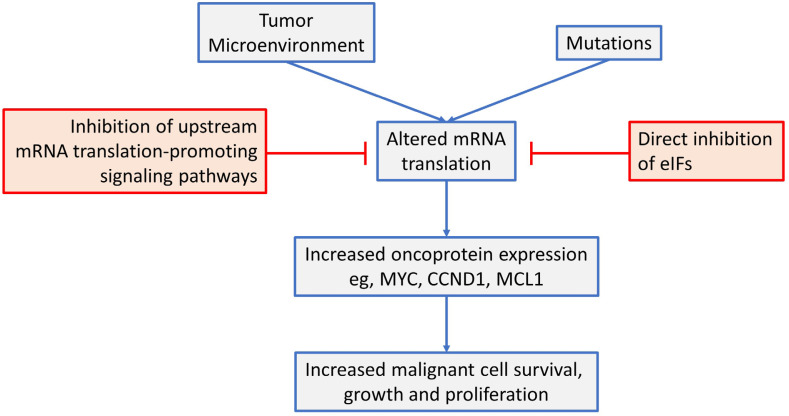
An overview of mRNA translation initiation dysregulation in B-cell neoplasms and its targeted inhibition. See text for details

This review has focused on dysregulated ofk mRNA translation in common B-cell neoplasms (see Figure 4 for a summary). Overall, there is now strong evidence from mouse models that altered mRNA translation contributes to lymphomagenesis and that both gene mutations and signals from the tumor microenvironment are important drivers in human neoplasms. These alterations in mRNA translation initiation can lead to increased expression of critical oncoproteins that support the growth, proliferation and survival of malignant B cells, such as MYC, MCL1 and cyclin D1. Moreover, targeted inhibition of either upstream, mRNA translation promoting pathways (e.g., by inhibition of mTORC1 or MNKs), or direct inhibition of eIFs themselves can lead to dramatic anti-tumor effects in preclinical models, *in vitro* and *in vivo*. Importantly, mRNA translationtargeting agents can be combined effectively with chemotherapy and other targeted agents (including kinase inhibitors and BCL2 inhibitors) and this is likely to be critical to boost responses and reduce opportunities for development of resistance.

Clinical testing has been most advanced for drugs targeted against mTORC1, which is a powerful upstream activator of mRNA translation. However, despite impressive preclinical results, this has not resulted in substantial clinical advances. It will be particularly interesting to see emerging results obtained with newer agents targeted against other mRNA translation promoting pathways (e.g., MNK inhibitors) or directly against eIFs which are approaching or have entered early phase clinical trials.

New biological insight will be critical to underpin further development of mRNA translation inhibition as a novel therapeutic strategy. This should encompass analysis of upstream activating pathways which driver mRNA translation dysregulation and downstream consequences for expression of specific effector proteins and cellular pathways. This will be key to determine how best to target mRNA translation in different tumor types to maintain strong anti-cancer activity whilst minimizing toxicity. It will also identify molecular biomarkers that are required for both patient selection and pharmacodynamics monitoring of patient responses, and to guide the development of rationale drug combinations to boost responses and reduce development of resistance.

## Abbreviations

4E-SE: 4E sensitivity element
ABC: activated B-cell-like
BCR: B-cell receptor
CERT: cytosine enriched regulator of translation
CHOP-R: cyclophosphamide, doxorubicin, vincristine, prednisone and rituximab
CLL: chronic lymphocytic leukemia
DLBCL: diffuse large B-cell lymphoma
eIF: eukaryotic initiation factor
FASN: fatty acid synthase
FCR: fludarabine, chlorambucil and rituximab
FL: follicular lymphoma
GC: germinal center
GCB: germinal center B-cell-like
Ig: immunoglobulin
IRES: internal ribosome entry site
M-CLL: mutated chronic lymphocytic leukemia
PABP: poly(A) binding protein
PIC: pre-initiation complex
SINEs: selective inhibitors of nuclear export
SLL: small lymphocytic lymphoma
TC: ternary complex
TOP: terminal oligopyrimidine
U-CLL: unmutated chronic lymphocytic leukemia
UTR: untranslated region

## Declarations

### Copyright

© The Author(s) 2020.
